# Probiotics for the management of irritable bowel syndrome: a systematic review and three-level meta-analysis

**DOI:** 10.1097/JS9.0000000000000658

**Published:** 2023-08-10

**Authors:** Min Chen, Lu Yuan, Chao-Rong Xie, Xiao-Ying Wang, Si-Jia Feng, Xin-Yu Xiao, Hui Zheng

**Affiliations:** a Department of Colorectal Diseases, Hospital of Chengdu University of Traditional Chinese Medicine; b Acupuncture and Tuina School, Chengdu University of Traditional Chinese Medicine, Chengdu, People’s Republic of China

**Keywords:** irritable bowel syndrome, multi-level meta-analysis, probiotics, systematic review

## Abstract

**Objective:**

Previous systematic reviews demonstrated a potentially beneficial effect of probiotics on irritable bowel syndrome (IBS). However, these studies are either affected by the inclusion of insufficient trials or by the problem of dependent data across multiple outcomes, and an overall effect size has not been provided. We aimed to determine the effect of probiotics on IBS through a three-level meta-analysis and clarify potential effect moderators.

**Methods:**

We searched MEDLINE, Embase, and Web of Science, screening for randomized controlled trials (RCTs) that examine the effect of probiotics on IBS. The primary outcome was the improvement in the severity of global IBS symptoms at the end of treatment. The secondary outcomes were the improvement in abdominal pain and the quality of life. The effect sizes of the probiotics were measured by using the standardized mean difference (SMD) and pooled by a three-level meta-analysis model.

**Results:**

We included 72 RCTs in the analysis. The meta-analysis showed significantly better overall effect of probiotics than placebo on the global IBS symptoms (SMD −0.55, 95% CI −0.76 to −0.34, *P*<0.001), abdominal pain (SMD −0.89, 95% CI −1.29 to −0.5, *P*<0.001) and quality of life (SMD 0.99, 95% CI 0.45 to 1.54, *P*<0.001), respectively. Moderator analysis found that a treatment duration shorter than 4 weeks was associated with a larger effect size in all the outcomes, and *Bacillus* probiotics had better improvement on the abdominal pain.

**Conclusions:**

Probiotics had a short-term effect and a medium effect size on the global IBS symptoms. Treatment duration and types of probiotics affected the effect size of probiotics, and shorter durations and *Bacillus* probiotics were associated with better treatment effects.

**Registration:**

Open Science Framework.

## Introduction

Highlights

- Despite the previous reports of several systematic reviews and meta-analyses examining the effect of probiotics for irritable bowel syndrome (IBS), the general effect size of probiotics on IBS symptoms and the essential effect moderators are unknown.
- In this meta-analysis incorporating 72 randomized controlled trials (RCTs), probiotics showed a medium effect size on the improvement of global IBS symptoms (standardized mean difference, −0.55, 95% CI −0.76 to −0.34) compared with placebo.
- A treatment duration shorter than 4 weeks and *Bacillus* probiotics were associated with larger effect sizes.

Irritable bowel syndrome (IBS) is a disorder of the brain–gut axis characterized by frequent abdominal pain, bloating, flatulence, and change of bowel habits – constipation or diarrhea. The global prevalence of IBS was 9.2% but varied across different regions; the prevalence was similar in Western countries, which is between 8.6 and 9.5% when the Rome III criteria were adopted and is between 4.5 and 4.7% with Rome IV^1^, and the prevalence was as high as 21.2% in Japan when the Rome III criteria were adopted^2^. IBS affects the quality of life substantially to the same degree as inflammatory bowel diseases^3^. The high prevalence and heavy disease burden urge the development of treatments for patients with IBS.

Owing to the complexity of IBS pathophysiology and the long disease duration, dietary supplements and alternative treatments are supposed to be more appropriate than pharmacological treatments since they are acknowledged to be harmless or with few adverse events. However, dietary supplements, like probiotics, are in the conundrum of ‘no harm, might help’^4^. Numerous systematic reviews and meta-analyses were published to examine the effect of probiotics on IBS, and most of them suggested a beneficial effect^5–8^, but convincing evidence cannot be reached owing to the small sample size, single-center design, and high risk of bias of the included trials. Additionally, the diverse outcome assessments and differential assessment time points hinder a general evaluation of the effect size of the probiotics. The previously published meta-analyses normally selected a specific time point and one of the outcomes to pool, which caused a loss of information – many of the outcomes are correlated and should be included for analysis^9^. In addition, numerous factors might affect the effect size of probiotics, and previous reviews concluded that specific strains of the probiotics had larger effects than the others^7^. Other effect moderators like treatment duration and the patient’s characteristics are rarely evaluated. Based on these grounds, we raised two clinically relevant questions – what is the overall effect size of probiotics in the management of IBS, and what are the major effect moderators that significantly affect the size of the probiotic effect?

One problem, not being fully settled in previously published meta-analyses on the topic, is that the effect sizes reported by the included trials might not be independent. For example, the studies conducted in the same region (i.e. European countries) might report similar results, which introduces dependence. A three-level meta-analysis is developed to solve this problem, which treats effect sizes nested within a study as dependent variables and examines the source of heterogeneity within a study and between studies – avoiding the inflation of type I error^10^. In addition, a three-level meta-analysis can include effect moderators in the model and assess the impact of the moderators on the effect sizes, which gives a better explanation for the effect of an intervention than the conventional meta-analysis. We aimed to assess the overall effect of probiotics on the improvement of IBS symptoms and find out the important effect moderators through the three-level meta-analysis.

## Methods

### Study overview

We performed a systematic review and multi-level meta-analysis, and this work had been reported in line with Preferred Reporting Items for Systematic Reviews and Meta-Analyses (PRISMA)^11^ (Supplemental Digital Content 1, http://links.lww.com/JS9/A874, Supplemental Digital Content 2, http://links.lww.com/JS9/A875) and AMSTAR (Assessing the methodological quality of systematic reviews) Guidelines^12^ (Supplemental Digital Content 3, http://links.lww.com/JS9/A876). The review was registered in Open Science Framework prior to conduction. The meta-analysis used aggregate-level data from published randomized controlled trials (RCTs). Ethical approvals and patient informed consent were acquired in each participating center of the RCTs. The work has been reported.

### Literature search and study selection

We searched MEDLINE, Embase, and Web of Science from inception to 12 November 2022, aiming to screen for RCTs that examined the efficacy of probiotics on IBS. Comprehensive search strategies with the combination of MeSH (Medical Subject Headings) terms and keywords were developed for the search in the databases. The search strategies were shown in eTables 1–3 (Supplemental Digital Content 4, http://links.lww.com/JS9/A877) We also searched previously published systematic reviews and read the reference lists of the reviews, trying to find out missing RCTs from our literature search. One author (C.-R.X.) performed the literature search, and two authors (L.Y. and X.-Y.X.) independently screened the retrieved articles. The inclusion and exclusion criteria are listed below.

RCTs were included if they (1) recruited participants who aged over 18 years and were diagnosed with IBS or any of its subtypes; (2) tested the effect of probiotics by comparing them with a placebo; (3) measured any of the following outcome: improvement of IBS symptoms, improvement of abdominal pain, or quality of life.

RCTs were excluded if they (1) also recruited other gastrointestinal diseases (e.g. functional dyspepsia, inflammatory bowel diseases); (2) published as letters that had insufficient information to judge the exact type of probiotics and their controls, or insufficient information on the types of outcomes.

### Outcome assessments

The primary outcome of this study was the severity of global IBS symptoms, which normally include abdominal pain, discomfort in the abdominal region (i.e. bloating, urgency, indigestion), and changed bowel habits (constipation, diarrhea, or diarrhea alternating with constipation). These symptoms could be assessed by asking questions with yes-or-no answers like ‘Are your global IBS symptoms relieved?’ or by adopting scales such as the IBS-SSS (Irritable Bowel Syndrome Severity Scoring System) scale.

Secondary outcomes included the severity of abdominal pain and the improvement in quality of life. Abdominal pain is one of the most essential symptoms of IBS, and it is usually separately reported. The severity of abdominal pain could be assessed by using binary outcomes indicating whether relief of pain was achieved or by rating scales such VAS (Visual Analog Scale) score. The quality of life in IBS patients is assessed by scales like IBS-QoL (Irritable Bowel Syndrome Quality of Life). Our study included all these scales.

### Data extraction

Two reviewers (X.-Y.W. and S.-J.F.) independently extracted study data from the included studies. Characteristics of the study design, participants, intervention, controls, and outcomes were separately extracted, and the characteristics were also coded and prepared for moderator analysis, which was described in detail in the statistical analysis section.

### Risk of bias assessment

We assessed the risk of bias (RoB) using the revised Cochrane RoB tool (RoB 2.0), in which five domains – bias arising from the randomization process, bias due to deviation from intended interventions, bias due to missing outcome data, bias in the measurement of an outcome, and bias in the selection of the reported result – were assessed and an overall RoB (low/high RoB or some concerns) was provided for each study. The certainty of the evidence was assessed by using the Grading of Recommendations, Assessment, Development, and Evaluations (GRADE) approach.

### Statistical analysis

We estimated the effect size of probiotics versus placebo by using the standardized mean difference (SMD, also known as Cohen’s *d*). Based on the assumption that the underlying continuous measurements in each intervention group follow a logistic distribution and that the variability of the outcomes is the same in both the intervention and control groups, the odds ratios (ORs) can be re-expressed as an SMD according to the following simple formula^13,14^: $\mathrm{SMD}=\;\frac{\surd 3}{\pi }\ln(\mathrm{OR})$ . The effect size was interpreted as small, medium, and large with the cut-off points of 0.2, 0.5, and 0.8, respectively^15^. To ensure the consistency of the direction of outcome measurements, the category outcomes were measured with the ORs of participants with failures of improvement, while continuous outcomes were measured with the change in the severity of the global IBS symptoms or abdominal pain.

We used a three-level random-effects meta-analysis model to pool the effect sizes, estimated the heterogeneity within-study (level 2) and between-study (level 3), and used Cochran’s *Q* test to evaluate whether the heterogeneity was statistically significant (defined as *P*<0.05). We compared the traditional two-level meta-analysis with the three-level model by evaluating the AIC (Akaike Information Criterion) and BIC (Bayesian Information Criterion) of the models and estimated the significance of the difference by the likelihood ratio test – which also used a cut-off point of 0.05. To assess potential publication bias, we generated funnel plots for the three outcomes to perform a visual assessment for asymmetry^16^.

We further performed moderator analyses to find out which factors substantially affected the effect size by using the meta-regression model. Four groups of factors were analyzed. The first group was study-design-related: countries hosting the studies, types of RCT (single vs. multicenter design), number of study sites, and the total study duration (measured by weeks). The second group was participant-related: age, proportion of females, the proportion of participants who dropped out from the study, duration of disease, and diagnostic criteria. The third group was intervention-related: duration of the intervention (measured by weeks) and the types of probiotics (classified as *Bacillus*, *Bifidobacterium*, *Enterococcus*, *Escherichia coli*, *Lactobacillus*, *Saccharomyces*, and a combination of differential probiotic strains). The fourth group was outcome-related: the type of data (continuous vs. categorical data) and types of outcome definition (i.e. global IBS symptoms could be further classified as adequate relief of symptoms, any relief of global symptoms, bloating, bowel habit, abdominal discomfort, and defecation urgency). The analysis was performed in the R environment (version 4.2.2) and the *metafor* package (version 4.2-0).

## Results

### Trial characteristics

**Figure 1:**
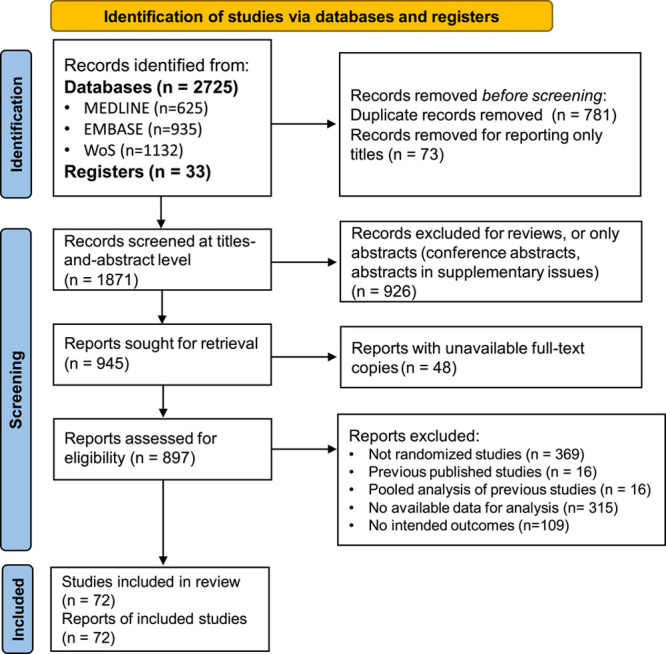
The study flowchart. WoS, Web of Science.

**Figure 2:**
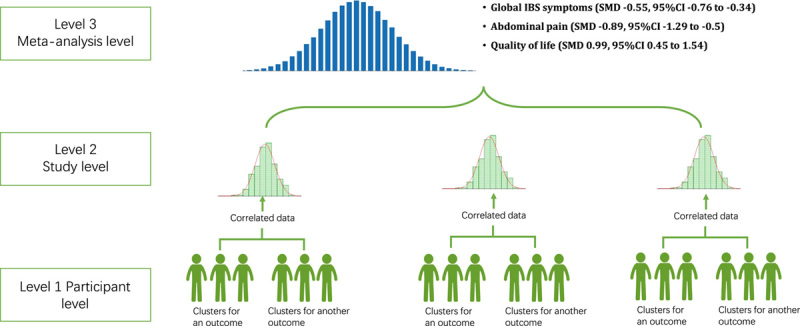
The three-level meta-analysis concept and results. IBS, irritable bowel syndrome; SMD, standardized mean difference. *Note*: The figure shows the concept of the three-level meta-analysis model and the results of the three outcomes. The three-level meta-analysis was conceived and developed to solve the problems of correlated data and missing information, which were unsatisfactorily settled in the traditional meta-analysis model^9^. For example, the severity of global IBS symptoms was assessed by differential scales at differential time points, and in most circumstances in traditional meta-analyses, data from one specific scale measured at one time point would be selected, which induced loss of information. We adopted the three-level model to synthesize all data from the relevant scales measured at defined time intervals, and we provided a general effect size using the SMD. According to previous literature, an absolute value of SMD larger than 0.5 would indicate a medium size of effect^15^, meeting the standard of recommendation for clinical practice. Our meta-analysis demonstrated that all SMDs for the three outcomes exceeded that cut-off point of 0.5.

We identified 72 RCTs^17–88^ after screening for 2725 pieces of articles and included a total of 8581 participants. The process of screening is shown in Figure 1. The included population had a mean age of 41.7 years and a mean proportion of 65.8% of females. Sixty-seven (93.1%) of the included studies adopted the Rome criteria as the diagnostic standard, and 53 (73.6%) of the studies recruited at least two subtypes of IBS. Of the 18 studies that focused on a single subtype, 13 studied diarrhea-predominated IBS. Seventy studies compared probiotics with placebo controls. More detailed information is shown in Table 1. Of the 72 included RCTs, nine were classified with a low risk of bias, two were with a high risk of bias, and 61 with some concerns; the assessment of each domain was presented in eFigure 1 (Supplemental Digital Content 4, http://links.lww.com/JS9/A877) The GRADE assessment for all three outcomes showed low certainty of the evidence. The summary of the GRADE table is shown in eTable 4 (Supplemental Digital Content 4, http://links.lww.com/JS9/A877). Figure 2 shows the concept of the three-level meta-analysis and the results of the meta-analyses on the three outcomes – the severity of global IBS symptoms, the severity of abdominal pain, and the quality of life assessment.

**Table 1:** Trial characteristics

Author^*^	Country, study design	Sample size (%female, mean age)	BMI	Group allocation (I, P)^†^	Diagnosis; Subtypes	The probiotics and their components^‡^	Dose and duration	Control, dosage and duration	The main efficacy outcomes
Gade et al.^17^	Denmark, RCT, Thirteen centers	54 (77.8, 34)	NA	32, 22	Others; IBS‑D, IBS‑C	*Enterococcus*; *Streptococcus faecium*	4 tablets b.i.d. for 4 weeks	Placebo; 4 tablets b.i.d.; 4 weeks	Improvement in IBS Symptoms
Nobaek et al.^18^	Sweden, RCT, Single center	60 (69.2, 48.5)	NA	25, 27	Rome criteria; IBS‑D, IBS‑C	*Lactobacillus*; *Lactobacillus plantarum* DSM 9843	400ml (5 × 10^7^ CFU/ml/drink) q.d.for 4 weeks.	Placebo; 400ml q.d.; 4 weeks.	Significant improvements in the IBS Symptoms
Niedzielin et al.^19^	Poland, RCT, Single center	40 (80,43.5)	23.7	20, 20	Clinical diagnosis; 2.5% IBS-D, 52.5% IBS-C, 45% IBS‑M	*Lactobacillus*; *Lactobacillus plantarum* 299V	200ml (5 × 10^7^CFU/ml)		
bid for 4 weeks	Placebo; 200ml bid; 4 weeks	Improvement in Global IBS symptoms							
Kim et al.^20^	USA, RCT, Single center	25 (72,42.8)	NA	12, 13	Rome II; 100% IBS‑D	Combination; VSL#3	One packet (containing 225 billion bacteria/packet) b.i.d. for 8 weeks	Placebo; One packet b.i.d.; 8 weeks	Satisfactory relief of IBS symptoms for 50% of weeks
Kim et al.^22^	USA, RCT, Single center	48 (93.8,43)	NA	24, 24	Rome II; 42 % IBS‑D, 33% IBS‑C, 25% IBS‑M	Combination; VSL#3	One product b.i.d. (31 patients for 4 weeks and 17 patients for 8 weeks)	Placebo; One product b.i.d. (31patients for 4 weeks and 17 patients for 8 weeks).	Satisfactory relief of IBS symptoms for 50% of weeks
Kajander et al.^21^	Finland, RCT, Single center	103 (76.5,45.5)	25.1	52, 51	Rome I and II; 47 .6% IBS‑D, 23.3% IBS-C, 29.1% IBS-M	Combination*Lactobacillus rhamnosus* GG, *Lactobacillus Rhamnosus* LC705, *Bifidobacterium breve* Bb99 and *Propionibacterium freudenreichii* ssp.	One capsule (8-9 × 10^9^ CFU/capsule) o.d. for 24 weeks	Placebo; one capsule o.d.; 24 weeks	Relief of global symptom score
Niv et al.^23^	Israel, RCT, Two centers	54 (66.7,45.7)	NA	27, 27	Rome II; 37% IBS‑D, 18.5% IBS-C 44.4% IBS‑M	*Lactobacillus*; *Lactobacillus reuteri* ATCC 55730	One product (1 × 10^8^colony-forming units/tablet), q.i,d. for 1week, then tid.	Placebo; One tablet b.i.d; 22 weeks.	Global symptoms score; Adverse events
Kim et al.^24^	Korea, RCT, Single center	40 (26.5,39.4)	23.7	20, 20	Rome II; 70 % IBS‑D, 30% IBS‑M	Combination; *Bacillus Subtilis* and *Streptococcus faecium*	One capsule t.i.d.; 4 weeks.	Placebo; One capsule t.i.d.; 4 weeks	Abdominal pain
Whorwell et al.^25^	UK, RCT, Twenty centers	362 (100,41.9)	26.7	270, 92	Rome II; 55.5% IBS-D, 20.7% IBS-C, 23.8% IBS-M,	*Bifidobacterium*; *Bifidobacterium infantis* 35624	(1 × 10^6^ live bacteria/capsule, 1 × 10^8^ live bacteria/capsule, or 1 × 10^10^ live bacteria/capsule) o.d. for 4 weeks	Placebo; One capsule o.d.; 4 weeks	Relief of overall IBS symptoms
Guyonnet et al.^26^	France, RCT, Thirty-five centers	274 (74.5,49.3)	NA	135, 132	Rome II; 100% IBS‑C	Combination; Fermented milk containing *Bifidobacterium animalis* DN1 73010 *Streptococcus thermophilus* and *Lactobacillus bulgaricus*	One pot containing 125g. (1.25 × 10^10^ CFU/125g) or (1.2 × 10^9^ CFU/125g) b.i.d. for 6 weeks	Placebo; One pot b.i.d.; 6 weeks	Improvement at least 10% discomfort dimension score
Drouault-Holowacz et al.^27^	France, RCT, Single center	106 (76,45.4)	NA	48, 52	Rome II; 29% IBS-D, 29% IBS-C, 41% IBS-M, 1% IBS-U	Combination; *Bifidobacterium longum* LA 101 (29%), *Lb.acidophilus* LA 102 (29%), *Lactobacillus lactis* LA 103 (29%), and *Streptococcus thermophilus* LA 104 (13%)	One sachet q.d. for 4 weeks	Placebo (of identical com-position except for the bacteria); One sachet q.d.; 4 weeks	Satisfactory relief of overall IBS symptoms
Enck et al.^28^	Germany, RCT, ten centers	297 (73.5,49.6)	24.2	149, 148	Clinical criteria; IBS-D, IBS-C, IBS-M	Combination; *Enterococcus faecalis* DSM16440 and *Escherichia coli* DSM17252	0.75 ml (3.0-9.0 × 10^7^ CFU/1.5ml) t.i.d. for 1 week, then 1.5 ml t.i.d. for weeks 2 and 3, then 2.25 ml t.i.d. for weeks 3–8	Placebo (identical in taste and Texture); The same dose; 8 weeks.	Have at least a 50% decrease in global symptom score
Kajander et al.^29^	Finland, RCT, Single center	86 (93,48)	26.2	43, 43	Rome II; 45% IBS-D, 25% IBS-C, 30% IBS-M	Combination; probiotic milk containing *Lactobacillus rhamnosus* GG (ATCC 53103, LGG), *Lactobacillus rhamnosus* Lc705 (DSM 7061), *Propionibacterium freudenreichii* ssp. *Shermanii* JS (DSM 7067) and *Bifidobacterium animalis ssp. lactis* Bb12 (DSM 15954)	One drink for 1.2 dL (1 × 10^7^ CFU/ml) q.d. for 14 weeks.	Placebo; One drink of 1.2 dL q.d. for 14 weeks.	Global IBS symptoms score
Sinn et al.^30^	Korea, RCT, Single center	40 (65,44.7)	22.2	20, 20	Rome III; 10% IBS‑D, 27 .5% IBS‑C, 62.5% IBS‑M	*Lactobacillus; Lactobacillus acidophilus*-SDC 2012 and 2013	One capsule (2 × 10^9^ CFU/ml) b.i.d. for 4 weeks	Placebo; One capsule b.i.d; 4 weeks.	Reduction in abdominal pain score
Zeng et al.^31^	China, RCT, Single center	30 (34.5,45.2)	NA	14, 15	Rome II; 100% IBS‑D	Combination; Fermented milk containing *Streptococcus thermophilus*, *Lactobacillus bulgaricus*, *Lactobacillus acidophilus*, and *Bifidobacterium longum*	200ml (1 × 10^8^ CFU/ml or 1 × 10^7^ CFU/ml) b.i.d.for 4 weeks	Placebo (containing no bacteria); 200mL b.i.d.; 4 weeks	Global IBS scores in GSRS
Agrawal et al.^32^	UK RCT, Single center	38 (100,39.5)	24.8	17, 17	Rome III; 100% IBS‑C	Combination; A fermented milk containing *Bifidobacterium lactis* DN-173010, *Streptococcus thermophilus*, and *Lactobacillus bulgaricus*	One pot (1.25 × 10^10^ CFU/ pot or 1.2 × 10^9^CFU/pot) q.d. for 4 weeks.	Placebo; One pot q.d.; 4 weeks.	Global symptoms score
Enck et al.^33^	Germany, RCT, Twelve centers	298 (49.3,49.6)	24,2	148, 150	Others; IBS‑D, IBS‑C, IBS‑M	*Escherichia coli; Escherichia coli* (DSM17252)	0.75ml (1.5‑4.5 × 10^7^ CFU/ml) drops t.i.d. for one week, then 1.5ml t.i.d. for weeks 2‑8	Placebo; 0.75ml drops t.i.d. for one week, then 1.5ml t.i.d. for weeks 2‑8	Adequate relief of IBS core symptoms
Hong et al.^34^	Korea, RCT, Single center	70 (32.9,37)	NA	36, 34	Rome III; 45.7% IBS-D, 20% IBS‑C, 8.5% IBS‑M,25.8% IBS‑U	Combination; *Bifidobacterium Bifidum* BGN4, *Bifidobacterium lactis* AD011, *Lactobacillus acidophilus* AD031, and *Lactobacillus casei* IBS041	One sachet (20 Billi bacteria/sachet) b.i.d. for 8 weeks	Placebo; One sachet b.i.d.;8 weeks	Reduction of symptom score by at least 50%
Hun et al.^35^	USA, RCT, Single center	50 (82,48)	NA	22, 22	Rome II;100% IBS-D	*Bacillus*; *Bacillus coagulans* GBI-30, 6086	One preparation q.d. for 8 weeks	Placebo; One preparation q.d.; 8 weeks	IBS symptoms (abdominal pain and bloating scores)
Williams et al.^36^	UK, RCT, Single center	56 (86.5,39)	NA	28, 28	Rome II; 11.5% IBS-D, 27% IBS-C, 61.5% IBS-M	Combination; *Lactobacillus acidophilus* CUL-60 (NCIMB 30157) and CUL‑21 (NCIMB 30156), *Bifidobacterium bifidum* CUL‑20 (NCIMB 30153), and *Bifidobacterium lactis* CUL‑34 (NCIMB 30172)	One capsule (2.5 × 10^10^ CFU/capsule) q.d. for 8 weeks	Placebo; One capsule q.d.; 8 weeks	IBS Symptom Severity Score
Simren et al.^37^	Sweden, RCT, Single center	74 (70.3,43)	NA	37, 37	Rome II; 35.1% IBS-D, 14.9% IBS‑C, 50% IBS‑M	Combination; Fermented milk containing *Lactobacillus paracasei*, *ssp. paracasei* F19, *Lactobacillus acidophilus* La5 and *Bifidobacterium lactis* Bb12	200 ml (5 × 10^7^ CFU/ml) b.i.d. for 8 weeks	Placebo; 200 ml nonfermented milk b.i.d.; 8 weeks	Adequate relief of IBS symptoms at least 50%
Choi et al.^38^	Korea, RCT, Three centers	90 (46,40.4)	23.1	45, 45	Rome II; 71.6 % IBS‑D, 28.4% IBS‑M	*Saccharomyces; Saccharomyces boulardii*	One capsule (2 × 10^11^ live cells/capsule) b.i.d. for 4 weeks	Placebo; One capsule b.i.d.; 4 weeks	Overall improvement in IBS-QOL
Guglielmetti et al.^39^	Germany, RCT,Multicenter	122 (67.2,38.9)	24.3	60, 62	Rome III; 21.3% IBS‑D, 19.7% IBS-C, 59% IBS‑M	*Bifidobacterium*; *Bifidobacterium Bifidum* MIMBb75	One capsule (1 × 10^9^CFU/capsule) q.d. for 4 weeks.	Placebo; One capsule q.d.; 4 weeks	Relief of overall IBS symptoms
Michail et al.^40^	USA, RCT, Single center	24 (66.7,21.8)	NA	15, 9	Rome III; 100% IBS-D	Combination; VSL#3	One packet (900 billion bacteria/packet) q.d for 8 weeks	Placebo; One packet q.d; 8 weeks	Global symptoms score (a clinical rating scale GSRS)
Ringel-Kulka et al.^41^	USA, RCT, Single center	33 (72,45.4)	NA	16, 17	Rome III; 100% IBS-D	Combination; *Lactobacillus acidophilus* NCFM (L-NCFM) and *Bifidobacterium animalis subsp. lactis* Bi-07 (B-LBi07)	(2 × 10^11^CFU/day) for 8 weeks	Placebo; 8 weeks	Global relief of GI symptoms, Satisfaction with treatment, HR-QOL
Sondergaard et al.^42^	Sweden, RCT, Two centers	64 (75,51.2)	24.8	27, 25	Rome II; Subtype not reported	Combination; Fermented milk containing *Lactobacillus paracasei ssp paracasei* F19, *Lactobacillus acidophilus* La5 and *Bifidobacterium lactis* Bb12	250ml (5 × 10^7^ CFU/ml) b.i.d. for 8 weeks.	Placebo; Acidified milk 250 ml b.i.d; 8 weeks	Adequate relief of IBS symptoms
Cha et al.^43^	Korea, RCT, Single center	50 (48,39.7)	23	25, 25	Rome III; 100% IBS-D	Combination; *Lactobacillus acidophilus, Lactobacillus plantarum, Lactobacillus rhamnosus, Bifidobacterium breve, Bifidobacterium lactis, Bifidobacterium longum*, and *Streptococcus thermophilus*	One capsule (0.5 × 10^10^ CFU/capsule) b.i.d. for 8 weeks.	Placebo; One capsule b.i.d.; 8 weeks.	Adequate relief of their IBS symptoms at least 50% of the weeks
Cui et al.^44^	China, RCT, Single center	60 (70,44.7)	21.2	37, 23	Rome III; 48.3% IBS-D, 30% IBS‑C, 11.7% IBS-M, 10% IBS-U	Combination; *Bifidobacterium longum* DSM 20219 and *Lactobacillus acidophilus* DSM 20079.	200mg t.i.d. for 4 weeks	Placebo; Two capsules t.i.d.; 4 weeks	Improvement in IBS symptoms
Dapoigny et al.^45^	France, RCT, Four centers	52 (70,47.1)	24	25, 25	Rome III; 30% IBS-D, 22% IBS-C, 34% IBS-M, 14% IBS-U	*Lactobacillus*; *lactobacillus casei rhamnosus* LCR35	One capsule 250 mg (2 × 10^8^ CFU/capsule) t.i.d. for 4 weeks	Placebo; 250 mg t.i.d.; 4 weeks	IBS severity Score
Ducrotte et al.^46^	France, RCT, Four centers	214 (29.4,37.3)	NA	108, 106	Rome III; 62% IBS-D, 38% non-classified	*Lactobacillus; Lactobacillus plantarum* 299V DSM 9843	One capsule (10 billion CFU/capsule) q.d. for 4 weeks.	Placebo; One capsule q.d.; 4 weeks	Relief of IBS Symptoms
Farup et al.^47^	Norway, RCT, Single center	16 (69,50)	24	/	Rome II; 37.5% IBS‑D 6.25% IBS‑C 56.25% IBS‑M	*Lactobacillus; Lactobacillus plantarum* MF 1298	One capsule (1 × 10^10^CFU/capsule) q.d. for 6 weeks.	Placebo; One capsule q.d.; 6 weeks.	Global symptoms score
Kruis et al.^48^	Germany, RCT, Single center	120 (76.7,45.7)	NA	60, 60	Rome II; 45% IBS-D, 29.2% IBS-C, 25.8%IBS-M/U	*Escherichia coli; Escherichia coli Nissle1917*	One capsule (2.5-25 × 10^9^ CFU/capsule) o.d. for 4 days then b.i.d. for 12 weeks	Placebo; The same protocol; 12 weeks	Clinical response (Patients Reported satisfied in treatment)
Murakami et al.^49^	Japan, Single center	35 (56.5,16.2)	NA	/	Rome III; subtype not reported	Lactobacillus; One capsule containing KB290 (freeze-dried KB290 bodies	(≥1.0 × 10^10^ CFU/capsule) q.d for 8 weeks	Placebo; One capsule q.d; for 8 weeks	Relief of IBS symptoms, Quality of life (QOL)
Begtrup et al.^50^	Denmark, RCT, Single center	131 (30,30.5)	24.5	67, 64	Rome III; 40.5% IBS‑D, 19.1% IBS‑C, 38.2% IBS‑M, 2.2% IBS‑U	Combination; *Lactobacillus paracasei ssp paracasei* F19, *Lactobacillus acidophilus La5, and Bifidobacterium Lactis Bb 12*	Two capsules (1.3 × 10^10^ CFU/capsule) b.i.d. for 6 months	Placebo; Two capsules b.i.d.; 24 weeks	Adequate relief of global IBS symptoms
Charbonneau et al.^51^	USA, RCT, Single center	76 (81.7,45.1)	30.2	39, 37	Rome II; subtype not reported	*Bifidobacterium; Bifidobacterium. infantis 35624*	One capsule (1 × 10^9^ CFU/capsule) q.d. for 8 weeks	Placebo; One capsule q.d.; 8 weeks.	Relief of IBS symptoms
Ko et al.^52^	Korea, RCT, Single center	26 (63.3,37.3)	22.8	13, 40	Rome III; 100% IBS‑D	Combination; *Bifidobacterium brevis, Bifidobacterium lactis, Bifidobacterium longum, Lactobacillus acidophilus, Lactobacillus plantarum, Lactobacillus rhamnosus*, and *Streptococcus thermophilus*	One capsule (5 billion bacteria/capsule) t.i.d. for 8 weeks.	Placebo; One capsule t.i.d.; 8 weeks.	Adequate relief of overall IBS symptoms
Roberts et al.^53^	UK, RCT, Thirteen centers	179 (85,44.2)	26.3	88, 91	Rome III; 100% IBS‑C or IBS‑M	Combination; *Bifidobacterium lactis* I-2494 (previously known as DN173010) *Streptococcus thermophilus I-1630, and Lactobacillus bulgaricus I‑1632 and I‑1519*	One pot (1.25 × 10^10^ CFU/pot or 1.2 × 10^9^ CFU/pot) b.i.d. for 12 weeks	Placebo;One pot b.i.d.; for 12 weeks	Subjective global assessment of symptom relief
Abbas et al.^54^	Pakistan, RCT, Single center	72 (26.4,35.4)	35.4	37, 35	Rome II; 100 % IBS‑D	*Saccharomyces; Saccharomyces boulardii*	750 mg q.d for 6 weeks (week 3-8)	Placebo; 6 weeks (week 3-8).	Abdominal pain
Jafari et al.^55^	Iran, RCT,Single center	108 (60.2,36.7)	NA	51, 46	Rome III; subtype not reported	Combination; *Bifidobacterium animalis subsp.* lactisBB‑12®, *Lactobacillus acidophilus* LA‑5®, *Lactobacillus delbrueckii subsp. bulgaricus* LBY‑27, and *Streptococcus thermophilus* STY‑31	One capsule (4 × 10^8^ CFU/capsule) b.i.d. for 4 weeks	Placebo; b.i.d.; 4 weeks	Relief of IBS symptoms
Lorenzo-Zuniga et al.^56^	Spain, RCT, Two centers	84 (63.1,46.8)	25.6	55, 29	Rome III; 100% IBS-D	Combination; *Lactobacillus plantarum* CECT7484, *Lactobacillus plantarum* CECT7485, and *Pediococcus acidilactici* CECT7483	High dose (1-3 × 10^10^ CFU/capsule) q.d. or Low dose (3-6 × 10^9^CFU/capsule) t.i.d. for 6 weeks	Placebo; One capsule q.d. for 6 weeks	Relief of IBS symptoms
Ludidi et al.^57^	Netherlands, RCT, Single center	40 (67.5,40.5)	25.5	21, 19	Rome III; 42.5% IBS‑D, 10% IBS‑C, 30% IBS‑M, 17 .5% IBS‑U	Combination; *Bifidobacterium lactis* W52, *Lactobacillus casei* W56, *Lactobacillus salivarius* W57, *Lactococcus lactis* W58, *Lactobacillus acidophilus* NCFM, and *Lactobacillus rhamnosus* W71	One sachet (5 × 10^9^ CFU/sachet) o.d. for 6 weeks	Placebo; One sachet (5 g) o.d. for 6 weeks	Mean symptom composite score
Pedersen et al.^58^	Denmark, RCT, Single center	123 (73.2,37.3)	22.7	41, 40	Rome III; 40.7% IBS-D, 15.4% IBS-C 38.2% IBS-M	*Lactobacillus*; *Lactobacillus rhamnosus* GG	One capsule b.i.d. for 6 weeks	Low FODMAP diet for 6 weeks; Normal Danish/Western diet for 6 weeks	Reduction of IBS-SSS
Rogha et al.^59^	Iran, RCT, Single center	85 (78.6,39.8)	NA	33, 39	Rome III; 12.5% IBS-C 32% IBS-D 50% IBS-M	*Bacillus*; *Bacillus* Coagulans and Fructo-oligosaccharides (100 mg).	One tablet (15 × 10^7^ Spores) t,i,d, for 12weeks	Placebo; One tablet t,i,d,; 12 weeks	Relief of IBS symptoms
Sisson et al.^60^	UK, RCT, Single center	186 (69.4,38.3)	NA	124, 62	Rome III; 37 .6% IBS‑D, 21.5% IBS-C, 35.5% IBS‑M, 5.4% IBS‑U	Combination; *Lactobacillus rhamnosus* NCIMB 30174, *Lactobacillus plantarum* NCIMB 30173, Lactobacillus *acidophilus* NCIMB 30175, and *Enterococcus faecium* NCIMB 30176	1 ml (1 × 10^10^ CFU/50 ml) q.d. for 12weeks	Placebo (containing inert flavorings and water); 1 ml q.d.; 12 weeks	IBS-SSS
Stevenson et al.^61^	South Africa, RCT, Single center	81 (97.5,47.9)	28.9	54, 27	Rome III; 37.6% IBS-D, 21.5% IBS-C	*Lactobacillus; Lactobacillus plantarum* 299v	Two capsules (5 × 10^9^ CFU/capsule) q.d. for 8 weeks	Placebo; Two capsules q.d.; 8 weeks	IBS symptom severity Scores
Yoon et al.^62^	Korea, RCT, Single center	49 (65.3,44.5)	NA	25, 24	Rome III; 53.1% IBS-D, 40.8% IBS-C, 6.1% IBS-M	Combination; *Bifidobacterium bifidum* KCTC 12199BP, *Bifidobacterium Lactis* KCTC 11904BP, *Bifidobacteriu Longum* KCTC 12200BP, *Lactobacillus acidophilus* KCTC 11906BP, *Lactobacillus rhamnosus* KCTC 12202BP, and *Streptococcus thermophilus* KCTC 11870BP	One capsule (5 × 10^9^viable cells/capsule) b.i.d. for 4 weeks	Placebo; One capsule b.i.d.; 4 weeks	Global relief of IBS Symptoms
Pineton de Chambrun et al.^63^	France, RCT, Single center	200 (86,44)	NA	93, 86	Rome III; 28.5% IBS-D, 46.9% IBS‑C, 24.6% IBS‑M	*Saccharomyces; Saccharomyces cerevisiae* CNCMI‑3856	One capsule (8 × 10^9^ CFU/capsule) q.d. for 8 weeks	Placebo; One capsule q.d.; 8 weeks	Improvement of abdominal pain adverse event
Wong et al.^64^	Singapore, RCT, Single center	42 (45.2,47)	NA	20, 22	Rome III; IBS‑M	Combination; VSL#3	Four capsules (225 billion bacteria/capsule) b.i.d. for 6 weeks	Placebo;four capsules b.i.d.; 6 weeks	Overall IBS symptom scores
Yoon et al.^65^	Korea, RCT, Single center	81 (46.3,59.3)	NA	39, 42	Rome III; 48.1% IBS-D, 18.5% IBS-C, 21%IBS-M, 12.4%IBS-U	Combination; *Bifidobacterium bifidum* (KCTC 12199BP,) *Bifidobacterium lactis* (KCTC11904BP), *Bifidobacterium Longum* (KCTC 12200BP), *Lactobacillus acidophilus* (KCTC 11906BP), *Lactobacillus rhamnosus* (KCTC 12202BP), and *Streptococcus thermophilus* (KCTC 11870BP)	One capsule (5 × 10^9^ viable cells/capsule) b.i.d. for 4 weeks.	Placebo; One capsule b.i.d.; 4 weeks	Adequate relief of overall IBS symptoms
Lyra et al.^66^	Finland, RCT, Two centers	391 (74.7,47.9)	24.7	260, 131	Rome III; 38.9% IBS‑D, 16.6% IBS‑C, 44% IBS‑M, 0.5% IBS‑U	*Lactobacillus; Lactobacillus acidophilus* NCFM (ATCC 700396)	One capsule (low dose: 1 × 10^9^ CFU/capsule; high dose: 1 × 10^10^ CFU/capsule) q.d. for 1 2 weeks	Placebo;One capsule q.d.; 12 weeks	IBS symptom severity Scores
Spiller et al.^67^	UK, RCT, Single center	379 (83.6,45.4)	NA	192, 187	Rome III; 20.8 % IBS-D, 47.5 % IBS‑C, 31.7% IBS‑M	*Saccharomyces; Saccharomyces. cerevisiae* I-3856	1000 mg (8 × 10^9^ CFU/g) q.d. for 12 weeks	Placebo (calcium phosphate and maltodextrin) q.d.; 12 weeks	Improvement of 50% of the weekly average "intestinal pain/discomfort score"
Thijssen et al.^68^	Holland, RCT, Four centers	80 (68.8,41.8)	25.1	39, 41	Rome II, 30% IBS-D, 25% IBS-C, 28.75% IBS-M, 16.25% IBS-U	A fermented milk drink (65ml per bottle); *Lactobacillus*	One bottle (6.5 × 10^9^ CFU/bottle) b.i.d. for 8 weeks	Placebo; One bottle (65ml) b.i.d.; 8 weeks	Mean symptom score decrease of at least 30%
Hod et al.^69^	Israel, RCT, Single center	107 (100,29.5)	22.3	54, 53	Rome III; 100% IBS-D	Combination; *Lactobacillus rhamnosus* LR5, *Lactobacillus casei* LC5, *Lactobacillus Paracasei.*LPC5, *Lactobacillus plantarum* LP3, *Lactobacillus acidophilus* LA1, *Bifidobacterium Bifidum* BF3, *Bifidobacterium Longum* BG7, *Bifidobacterium breve* BR3, *Bifidobacterium infantis* BT1, *Streptococcus, thermophilus* ST3, *Lactococcus bulgaricus* LG1, *and Lactococcus Lactis* SL6	b.i.d. for 8 weeks	Placebo; b.i.d.; 8 weeks	Abdominal pain, overall responder rates
Pinto-Sanchez et al.^70^	Canada, RCT, Single center	44 (54,43.3)	24.9	22, 22	Rome III; 61.4% IBS‑D, 38.6% IBS‑M	*Bifidobacterium; Bifidobacterium longum*, NCC3001	(1.0 × 10^10^ CFU/gram powder with maltodextrin) for 6 weeks.	Placebo; 6 weeks	Adequate relief of IBS symptoms
Staudacher et al.^71^	UK, RCT, two centers	104 (67.5,35.5)	24.5	/	Rome III; 66.3% IBS-D, 23.1% IBS-M, 10.6% IBS-U	Combination; VSL#3	4 weeks	Placebo, sham diet; 4 weeks	Adequate relief of IBS symptoms
Shin et al.^72^	Korea, RCT, Single center	60 (56.9,36.5)	23.4	27, 24	Rome III; 100% IBS‑D,	*Lactobacillus; Lactobacillus. gasseri* BNR17	Two capsules b.i.d. for 8 weeks	Placebo; Two capsules b.i.d.; 8 weeks	Relief of IBS symptoms
Catinean et al.^73^	Romania, RCT, Single center	90 (60,39.4)	25.2	30, 60	Rome III; 100 % IBS-D	*Bacillus;* MegaSporeBiotic a mixture of spores of five *Bacillus spp*	q.d. for 1 week, then b.i.d. for 24 days.	Ten-days rifaximin treatment followed by either a nutraceutical agent or a Low FODMAPs for 24 days	IBS-SSS Score
Madempudi et al.^74^	India, RCT, Single center	136 (27.8,43.4)	24.8	53, 55	Rome III; IBS-D, IBS‑C, IBS‑M, IBS‑U	*Bacillus*; *Bacillus* coagulants Unique IS2	One capsule (2 billion CFU/capsule) q.d. for 8 weeks	Placebo;One capsule q.d.; 8 weeks	Relief of abdominal pain, Satisfactory relief of IBS symptoms
Oh et al.^75^	Korea, RCT, Single center	55 (72,32.8)	21.5	26, 24	Rome III; 42% IBS‑D,20% IBS‑M, 38% IBS‑U	Combination; *Lactobacillus species, Lactobacillus paracasei, Lactobacillus salivarius*, and *Lactobacillus plantarum.*	q.d. for 4 weeks.	Placebo; q.d.; 4 weeks.	Relief of IBS Symptoms
Andresen et al.^76^	Germany, RCT, twenty-center	443 (69.5,41.4)	24.6	221, 222	Rome III; 40% IBS‑D, 24.1% IBS‑C, 7.7% IBS‑M, 28.2% IBS‑U	*Bifidobacterium*; *Bifidobacterium bifidum* MIMBb75	Two capsules (1 × 10^9^ cells/capsule) q.d. for 8 weeks	Placebo; q.d.; 8 weeks	Adequate relief of IBS symptoms
Gayathri et al.^77^	India, RCT, Single center	100 (34,41)	NA	52, 48	Rome III;65 % IBS-D, 24 % IBS‑C, 11% IBS‑M	*Saccharomyces*; *Saccharomyces cerevisiae* CNCM I-3856	One capsule (2 × 10^9^ CFU/capsule) b.i.d. for 8 weeks	Placebo; One capsule b.i.d.; 8 weeks.	Abdominal pain score
Kim et al.^78^	Korea, RCT, Single center	63 (74.6,36)	NA	32, 31	Rome II; 100% IBS-D	Combination; *Bifidobacterium* longum BORI, *Bifidobacterium bifidum* BGN4, *Bifidobacterium lactis* AD011, *Bifidobacterium infantis* IBS007, and *Lactobacillus acidophilus* AD031	One capsule (5 × 10^9^viable cells/capsule) t.i.d. for 8 weeks	Placebo; One capsule t.i.d.; 8 weeks	Relief of IBS symptoms
Lewis et al.^79^	Canada, RCT, Single center	285 (77.7,42)	NA	167, 81	Rome III; 15.1% IBS-D, 11.2% IBS-C, 73.7%IBS-M	*Lactobacillus* or *Bifidobacterium*; *Lactobacillus paracasei* HA-196 or *Bifidobacterium longum* R0175	One capsule (10 × 10^9^ CFU/capsule) q.d. for 8 weeks.	Placebo; One capsule q.d.; 8 weeks.	IBS Symptom Severity Score
Martoni et al.^80^	USA, RCT, Twelve-center	336 (49.5,39.5)	24	224, 112	Rome III; Subtype not reported	*Lactobacillus* or *Bifidobacterium; Lactobacillus acidophilus* DDS-1 or *Bifidobacterium lactis* UABla‐12	One capsule (1 × 10^10^ CFU/capsule) q.d. for 6 weeks.	Placebo; q.d.; 6 weeks	Relief of IBS symptoms score
Sadrin et al.^81^	France, RCT, Multicenter	80 (71,48.9)	NA	40, 40	Rome III; Subtype not reported	*Lactobacillus; Lactobacillus acidophilus* NCFM *and Lactobacillus acidophilus subsp. Helveticus* LAFTI L10	Two capsules (5 × 10^9^CFU/capsule) b.i.d. for 8 weeks	Placebo;Two capsules b.i.d.; 8 weeks	Relief of IBS symptoms score
Wilson et al.^82^	UK, RCT, Single center	69 (55.1,34.1)	24.7	24, 45	Rome III; 65% IBS-D	*Bifidobacterium; Bifidobacterium bifidum* NCIMB 41171	q.d. for 4 weeks.	Placebo; q.d.; 4 weeks.	Relief of IBS Symptoms
Barraza-Ortiz et al.^83^	México, RCT, Single center	55 (67.2,45.5)	25.2	37, 18	Rome IV; 52.7% IBS-D 47.3% IBS-M	Combination; *Lactobacillus plantarum* CECT 7484, *Lactobacillus plantarum* CECT 7485, and *Pediococcus acidilactici* CECT 7483	t.i.d for 6 week	Placebo; t,i,d, 6 weeks	Response rate in QoL, Abdominal pain
Gupta et al.^84^	India, RCT, Single center	40 (30,35.5)	23.8	20, 20	Rome IV; Subtype not reported	*Bacillus*; *Bacillus* coagulants LBSC	t.i.d for 80 days	Placebo; t,i,d,; 80 days	IBS-SSS, Change in stool consistency
Skrzydło-Radomańska et al.^85^	Poland, RCT, Single center	51 (64.5,43.1)	25.9	25, 23	Rome III; 100% IBS-D	Combination; *Bifidobacterium*, *Lactobacillus*, and *Streptococcus thermophilus*	One capsule b.i.d. for 8 weeks	Placebo; One capsule b.i.d.; 8 weeks	IBS-SSS, Global Improvement Scale (IBS-GIS)
Mack et al.^86^	Germany, RCT, Multicenter	389 (69,46.4)	26	191, 198	Rome III; 16.1% IBS-C 39.8% IBS-D 36,2% IBS-M	Combination; *Escherichia coli* (DSM 17252) and *Enterococcus faecalis* (DSM 16440)	10 drops (0.71 mL) 3 × /day during week 1, 20 drops (1.42 mL) 3 × /day during week 2, 30 drops (2.14 mL) 3 × /day during week 3, and 30 drops 3 × /day until week 26	Placebo; The same dose and duration	IBS Global Assessment of Improvement Scale (IBS-GAI), Abdominal pain
Moeen-Ul-Haq et al.^87^	Pakistan, RCT, Single center	120 (41.7,35.9)	NA	55, 53	Rome III; 25.9% IBS-C 30.6% IBS-D 43,5% IBS-M	*Lactobacillus*; *Lactobacillus plantarum* 299v	(5x10^10^ CFU) for 4 weeks	Placebo (comprised micro-crystalline cellulose powder); 4 weeks	Daily frequency of abdominal pain, Improvement in the severity of abdominal pain, The severity of bloating
Mourey et al.^88^	France, RCT, Four centers	456 (86,40.5)	NA	230, 226	Rome IV; 100% IBS-C	*Saccharomyces*; *Saccharomyces cerevisiae* CNCM I-3856	Two capsules (8 × 10^9^ CFU/capsule) q.d. for 8 weeks	Placebo; q.d.; 8 weeks	Global IBS symptoms

BMI, body mass index; B.i.d, twice a day. CFU, colony forming units; FODMAP, fermentable oligosaccharides, disaccharides, monosaccharides and polyols; GSRS, gastrointestinal symptom rating scale; IBS, irritable bowel syndrome; IBS-C, constipation-predominant irritable bowel syndrome; IBS-D, diarrhea-predominant irritable bowel syndrome; IBS-M, mixed irritable bowel syndrome; IBS-U, un-subtyped irritable bowel syndrome; IBS-SSS, irritable bowel syndrome symptom severity score; IBS-QOL, evaluation of the irritable bowel syndrome quality of life; O.d, once every two days. Q.d, four times a day; RCT, randomized controlled trial; T.i.d, three times a day. VSL#3, a combination of three types of *Bifidobacterium* (*Bifidobacterium longum*, *Bifidobacterium infantis*, and *Bifidobacterium breve*).

* This column is arranged according to years.

† (I, P) refers to the sample sizes for probiotics and control groups, respectively, whereas the I refers to the probiotic group and the P refers to the control group.

‡ This column lists numerous abbreviations for the probiotic components which were named as the article reported or according to the nomenclature or naming system for bacteria suggested by the International Committee on Systematics of Prokaryotes.

### The severity of global IBS symptoms

The three-level meta-analysis – included 63 studies^17–22,25–34,36–48,50,52,53,55–58,60–69,71–76,78–81,83–87^ and generated 217 effect sizes – showed an overall effect of probiotics was significantly superior over placebo (SMD −0.55, 95% CI −0.76 to −0.34, *P*<0.001; Fig. 2), but with significant heterogeneity (Cochran’s *Q* =2906.24, *P*<0.001). We further analyzed within-study and between-study variance and found a total *I* ^2^ of 96.3%, a within-study *I* ^2^ of 79.5%, and a between-study *I* ^2^ of 16.8%. We then compared the traditional two-level meta-analysis model with the three-level model and found the three-level model reduced AIC from 913.7 to 906 and the BIC from 920.4 to 916.2, and the likelihood ratio test (LRT) demonstrated a significant difference between the two models (chi-squared (*χ* ^2^) =9.652, *P*=0.002) – showing the three-level model provided a better fit. The funnel plot showed no signs of publication bias (eFigure 2, Supplemental Digital Content 4, http://links.lww.com/JS9/A877).

**Figure 3:**
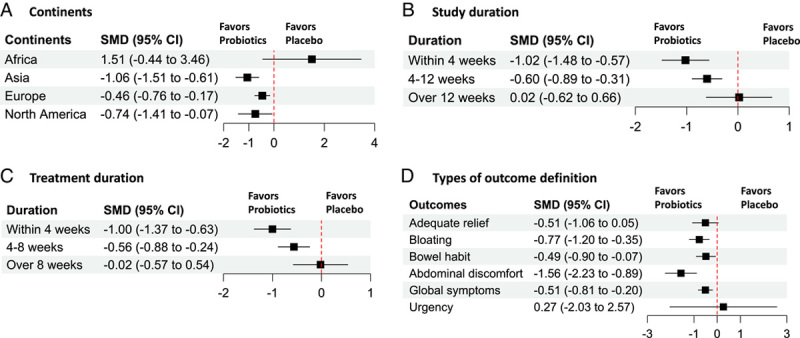
The moderators of the probiotic effects on global IBS symptoms. IBS, irritable bowel syndrome. SMD, standard mean difference. *Note*: The figure shows that (A) continents, (B) study duration, (C) treatment duration, and (D) types of outcome definition are the most important moderators of the probiotic effects. (A) The study population in Asia, Europe, and North America had larger effect sizes than in Africa. (B) and (C), shorter study duration (<4 weeks) and treatment duration (<4 weeks) were associated with larger effect sizes. (D) The outcome of abdominal discomfort was associated with larger effect sizes than other outcomes.

The moderator analyses showed that participants from different continents reported differential effects, while participants from the Asia region reported the largest effect size (Fig. 3A). Additionally, we found that study duration affected the effect size, and a longer study duration was associated with a smaller effect size (Fig. 3B). We also found that longer treatment duration was associated with a smaller effect size (Fig. 3C). The type of outcome impact also had an impact, and probiotics had a larger effect size on abdominal discomfort (SMD −1.55, 95% CI −2.97 to −0.15, *P*=0.017; Fig. 3D). Other factors had no significant impact on the effect sizes of the probiotics (Table 2).

**Table 2:** Moderator analysis of the outcome measurements.

	Global IBS symptoms		Abdominal pain		QoL assessment	
Moderators	Estimate	*P* ^*^	Estimate	*P* ^*^	Estimate	*P* ^*^
Continents						
Africa	1.51 (−0.44 to 3.46)	Reference	NA	NA	−0.23 (−3.38 to 2.92)	Reference
Asia	−1.06 (−1.51 to −0.61)	0.012	−1.44 (−2.24 to −0.64)	Reference	2.71 (0.72 to 4.7)	0.118
Europe	−0.46 (−0.76 to −0.23)	0.05	−0.91 (−1.58 to −0.23)	0.314	0.98 (−0.07 to 2.02)	0.466
North American	−0.74 (−1.41 to −0.07)	0.033	−0.2 (−1.78 to 1.38)	0.167	0.76 (−0.99 to 2.52)	0.58
Assessment time points						
Within 8 weeks	−0.7 (−0.93 to −0.48)	Reference	−0.97 (−1.36 to −0.59)	Reference	1.18 (0.56 to 1.79)	Reference
9–16 weeks	0.06 (−0.46 to 0.57)	0.05	−0.85 (−1.79 to 0.08)	0.807	0.53 (−0.28 to 1.34)	0.128
17–24 weeks	−0.26 (−1.1 to 0.59)	0.316	−0.54 (−2.62 to 1.53)	0.686	0.65 (−1.9 to 3.19)	0.68
>24 weeks	1.48 (−0.89 to 3.86)	0.07	NA	NA	NA	NA
RCT types						
Single center	−0.66 (−0.96 to −0.37)	0.672	−1.15 (−1.74 to −0.55)	Reference	1.3 (0.1 to 2.49)	Reference
Multicenter	−0.55 (−0.97 to −0.14)	0.614	−0.82 (−1.68 to 0.04)	0.534	0.99 (−0.16 to 2.15)	0.715
Multi-nation	0.18 (−2.67 to 3.04)	Reference	NA	NA	NA	NA
Number of study sites	0.02 (−0.02 to 0.04)	0.375	0.04 (−0.034 to 0.107)	0.305	−0.03 (−0.28 to 0.22)	0.824
Study duration						
<4 weeks	−1.02 (−1.48 to −0.57)	Reference	−1.71 (−2.62 to −0.8)	Reference	6.58 (4.09 to 9.07)	Reference
4–12 weeks	−0.6 (−0.89 to −0.31)	0.126	−0.75 (−1.37 to −0.13)	0.086	0.95 (0.31 to 1.58)	<0.001
>12 weeks	0.02 (−0.62 to 0.66)	0.009	−0.94 (−2.45 to 0.57)	0.387	0.54 (−0.27 to 1.35)	<0.001
Age	0.02 (−0.02 to 0.06)	0.44	−0.006 (−0.1 to 0.09)	0.895	−0.25 (−0.51 to 0.006)	0.055
Proportion of female	0.005 (−0.008 to 0.02)	0.463	0.02 (0.001 to 0.046)	0.04	−0.05 (−0.1 to 0.008)	0.088
Proportion of drop-outs	0.008 (−0.004 to 0.02)	0.205	0.02 (−0.02 to 0.05)	0.347	−0.007 (−0.05 to 0.03)	0.748
Disease duration	0.03 (−0.06 to 0.122)	0.523	0.11 (−0.78 to 1)	0.791	−0.11 (−0.62 to 0.39)	0.635
Diagnostic criteria						
Others	−0.78 (−1.78 to 0.25)	Reference	−1.07 (−2.82 to 0.67)	Reference	1.18 (−3.03 to 5.4)	Reference
Rome	−0.25 (−2.4 to 1.91)	0.667	−0.39 (−3.42 to 2.65)	0.697	NA	NA
Rome II	−0.61 (−1.1 to −0.16)	0.784	−1.37 (−2.41 to −0.32)	0.774	1.55 (0.23 to 2.87)	0.866
Rome III	−0.62 (−0.94 to −0.3)	0.781	−1 (−1.65 to −0.35)	0.936	0.85 (−0.46 to 2.16)	0.88
Rome IV	−0.56 (−1.91 to −0.79)	0.807	−0.38 (−2.58 to 1.83)	0.623	0.33 (−3.13 to 3.79)	0.753
Treatment duration						
<4 weeks	−1 (−1.37 to −0.63)	Reference	−1.45 (−2.24 to −0.65)	Reference	6.58 (4.13 to 9.02)	Reference
4–8 weeks	−0.56 (−0.88 to −0.24)	0.08	−0.89 (−1.56 to −0.22)	0.286	0.99 (0.44 to 1.54)	<0.001
>8 weeks	−0.02 (−0.57 to 0.54)	0.004	−0.26 (−1.87 to 1.35)	0.19	0.1 (−0.94 to 1.14)	<0.001
Types of probiotics						
*Bacillus*	−0.58 (−1.62 to 0.46)	Reference	−2.23 (−4.31 to −0.14)	Reference	NA	NA
*Bifidobacterium*	−0.77 (−1.42 to −0.12)	0.757	−0.44 (−1.54 to 0.65)	0.136	0.77 (−0.27 to 1.81)	Reference
Combination	−0.65 (−0.96 to −0.34)	0.901	−1.24 (−1.85 to −0.63)	0.37	1.18 (0.65 to 1.71)	0.487
*Enterococcus*	−0.51 (−2.07 to 1.06)	0.939	−1.38 (−4.44 to 1.69)	0.651	NA	NA
*Escherichia coli*	−0.59 (−2.06 to 0.87)	0.987	−0.65 (−3.97 to 2.67)	0.427	0.61 (−0.92 to 2.15)	0.866
*Lactobacillus*	−0.6 (−1.07 to −0.13)	0.969	−1.04 (−1.78 to −0.31)	0.29	0.12 (−0.49 to 0.73)	0.285
*Saccharomyces*	−0.09 (−1.21 to 1.03)	0.529	0.41 (−0.93 to 1.75)	0.037	6.45 (4.36 to 8.55)	<0.001
Types of outcomes						
Continuous	−0.5 (−0.82 to −0.18)	Reference	−1.08 (−1.55 to −0.6)	Reference	1.4 (0.56 to 2.24)	Reference
Categorical	−0.71 (−0.97 to −0.44)	0.301	−0.59 (−1.37 to 0.18)	0.279	1.23 (0.06 to 2.4)	0.895
Types of outcome definition			NA	NA	NA	NA
Adequate relief	−0.51 (−1.06 to 0.05)	Reference				
Bloating	−0.77 (−1.2 to −0.35)	0.445				
Bowel habits	−0.49 (−0.9 to −0.07)	0.953				
Abdominal discomfort	−1.56 (−2.23 to −0.89)	0.017				
Global symptoms	−0.51 (−0.81 to −0.2)	0.999				
Urgency	0.27 (−2.03 to 2.57)	0.518				

NA, not available; QoL, quality of life.

* The *P* values were estimated as the reference categories being compared with the reference category.

### The severity of abdominal pain

The model – included 48 studies^17–22,24–34,36,37,39,40,43–46,49,52,54,55,57,62–67,69,72,74,75,77–81,84,87,88^ and generated 92 effect sizes – showed probiotics reduces the severity of abdominal pain (SMD −0.89, 95% CI −1.29 to −0.5, *P*<0.001; Fig. 2), but with significant heterogeneity (Cochran’s *Q* =1923.12, *P*<0.001). We found a total *I* ^2^ of 98.4%, a within-study *I* ^2^ of 74.7%, and a between-study *I* ^2^ of 23.7%. A slightly reduced AIC (from 363 to 360.5) was found in the three-level model when compared with the traditional one, the LRT test was still significant (*χ* ^2^=4.1825 *P*=0.034). The funnel plot showed no signs of publication bias (eFigure 3, Supplemental Digital Content 4, http://links.lww.com/JS9/A877).

In the moderator analysis, we found that the study duration affected the effect sizes. RCTs with a study duration shorter than 4 weeks (SMD, −1.71, 95% CI −2.62 to −0.8) had significantly larger effect sizes than other RCTs with a study duration longer than 4 weeks (*P*<0.001; Table 2). The proportion of females also affected the effect sizes, RCTs with a higher proportion of females were associated with smaller effect sizes (coefficient estimate 0.02, 95% CI 0.001–0.046, *P*=0.04). For other factors, no significant impact was found (Table 2).

### Quality of life assessment

The model – included 23 studies^20,22,23,28,33,36–38,40,43,48,56,60,61,63,66,68,71,78,80,83,85,88^ and generated 71 effect sizes – showed that probiotics significantly improved the quality of life in patients with IBS (SMD 0.99, 95% CI 0.45–1.54, *P*<0.001; Fig. 2), but with significant heterogeneity (Cochran’s *Q* =788.7, *P*<0.001). The total *I* ^2^, within-study *I* ^2^, and between-study *I* ^2^ were 98%, 41.8%, and 56.2%, respectively. Compared with the traditional meta-analysis model, the three-level model had significantly reduced AIC (268.2–257.5) and BIC (272.7–264.2) (LRT=12.7, *P*<0.001). The funnel plot showed signs of publication bias (eFigure 4, Supplemental Digital Content 4, http://links.lww.com/JS9/A877); one study^37^ demonstrated a larger size than other studies.

The moderator analysis showed that the treatment duration and the types of probiotics affected the effect size. A treatment duration within 4 weeks showed a significantly larger effect than a treatment duration between 4 and 8 weeks and a treatment duration longer than 8 weeks (Table 2). The probiotic strains containing *Bacillus* and *Bifidobacterium* showed significantly larger effect sizes than those containing *Saccharomyces* (Table 2).

## Discussion

By pooling all the effect sizes from the included RCTs, we found a general medium effect size (with an SMD larger than 0.5) of probiotics on the improvement of IBS symptoms compared with placebo, and a large effect size (with an SMD larger than 0.8) of probiotics on the abdominal pain and the scores of quality-of-life assessments. We found that the treatment duration and study duration were the most important moderators of effect, and a longer study duration or treatment duration was associated with a smaller effect size. When the treatment duration was longer than 8 weeks and the study duration was longer than 12 weeks, the effect sizes dropped to −0.02 and 0.02 (extremely small effect size), respectively.

Our meta-analysis included a larger number of studies than the previous and recent systematic reviews that assessed the efficacy of probiotics for IBS^6,7^ because we used the transformation between odds ratios and SMDs, which has been suggested and reported in the Cochrane handbook^16^ and methodological reports^9,14^ to increase the statistical strength of the meta-analysis. The ability to include more studies might also be attributed to the application of the three-level meta-analysis model, which prompts the estimation of the general effect of the probiotics on IBS and confirms a medium effect size of probiotics on the improvement of global IBS symptoms (SMD 0.56) – suggesting a possible generalization to routine practice.

Shorter treatment duration or study duration being associated with larger treatment effects of probiotics was one of the major findings of our meta-analysis. A network meta-analysis published in 2022 reported that treatment duration could affect the efficacy of probiotics in the relief of abdominal pain and strain, and it showed that using *Bacillus coagulans* for 8 weeks was the most efficacious^89^. Dale and colleagues found that longer treatment duration might be associated with better efficacy in the treatment of IBS with probiotics^90^, while the other two studies showed that a shorter treatment duration of probiotics would be more efficacious^91,92^. Our meta-analysis, with a larger number of included trials and the inclusion of more efficacy data (through a third-level model), confirmed that shorter treatment duration was associated with a larger treatment effect. Several hypotheses were proposed for this phenomenon. First, trials with small sample sizes and single-center design were more likely to have short treatment duration and study duration, which could be the consequence of a shortage of study funding, so the larger treatment effects could be explained by small-study effects^93^. Second, the larger effect of probiotics on IBS might also be attributed to a powerful placebo – similar to the effect of sham device or sham acupuncture reported in previous studies^94–96^. In a meta-analysis investigating the magnitude of placebo response in IBS trials, short treatment duration was found to be associated with a large placebo effect^97^. Third, the moderator effect of short treatment duration might reflect the association between the severity of the IBS symptoms and the effect of the assessed intervention. Patients with severer IBS symptoms might report a smaller treatment effect of probiotics, and the physicians might be inclined to suggest a longer treatment duration, especially for those with refractory IBS^98^. Regarding that, the diagnostic criteria of refractory IBS are difficult to define and the severity of IBS disease is determined by several factors – health-related quality of life, psychosocial factors, healthcare utilization behaviors, and burden of illness^99^, so it is impossible to test this hypothesis based on the included trials in this meta-analysis since most of the included studies did not classify the disease severity owing to the lack of standard criteria. This informs that there is an urgent need for a standard scale to estimate the overall severity of IBS to minimize the heterogeneity caused by the study population in future meta-analyses on probiotics for IBS. Additionally, future RCTs are encouraged to report symptom severity of IBS using scales like IBS-SSS in baseline evaluation to facilitate subgroup or meta-regression analysis for clarifying the relationship between severity of IBS and probiotic treatment duration.

Although we did not find an impact of different types of probiotics on the improvement of IBS symptoms, we found that *Bacillus* strains led to better improvement in abdominal pain than other strains and were significantly better than the *Saccharomyces* strain. This finding was consistent with a recent network meta-analysis comparing differential probiotics for the treatment of IBS^7^, which implies that the *Bacillus* strains might be developed for the treatment of functional abdominal pain and warrants further clarification.

Our study had several limitations. First, the certainty of the evidence was low because most of the included trials were classified as with some concerns or a high risk of bias. Many trials had some concerns in the randomization process (mainly the problem of the transparency of allocation concealment) and the measurement of the outcomes. Second, the large heterogeneity in the meta-analysis was also a concern. The heterogeneity might be caused by the difference in the study population and the intervention protocols. We ran the moderator analysis and confirmed that the duration of treatment and study, the study regions, and the types of outcomes might be the source of heterogeneity. Third, although the method of transforming between OR and SMD enlarged the sample size of the meta-analysis, it made the explanation of the results difficult for clinical practitioners, who might transform it back to the original scale by multiplying the SMD generated from the meta-analysis by the standard deviation of the specific scale^14^. Fourth, forty-eight reports were excluded for the unavailability of full-text copies, which were mainly abstracts of conference presentations and supplementary issues. These reports, known as grey literature, might be valuable for our meta-analysis and might change the conclusion of our study. Updated systematic review and meta-analysis might therefore be warranted while many of them were available with sufficient data for analysis.

## Conclusions

Our meta-analysis suggested a medium short-term effect of probiotics on the improvement of global IBS symptoms and abdominal pain. We found that the treatment duration, study regions, the types of outcomes, and the types of probiotics might be major effect moderators, which warrants further investigation.

## Ethical approval

This article is a systematic review and meta-analysis; ethical approvals were acquired in the original studies, and additional approval was not required for this meta-analysis.

## Consent

This article is a systematic review and meta-analysis; consents were acquired in the original studies, and additional consents were not required for this meta-analysis.

## Sources of funding

M.C. received a grant from the National Natural Science Foundation of China (No. 82274529) and a grant (no. 2022NSFSC1503) from the Science and Technology Department of Sichuan Province. H.Z. received a grant from the Sichuan Youth Science and Technology Innovation Research Team (no. 2021JDTD0007).

## Author contribution

All authors had full access to all the data in the study and take responsibility for the integrity of the data and the accuracy of the data analysis. M.C. and H.Z.: conceived and designed the study; M.C., L.Y., C.-R.X., X.-Y.W., S.-J.F., and X.-Y.X.: acquired, analyzed, and interpreted the study data. M.C.: drafted the manuscript. All authors revised the manuscript.

## Conflicts of interest disclosure

The authors declare that they have no conflicts of interest.

## Research registration unique identifying number (UIN)

Open Science Framework (https://osf.io/rq87j).

## Guarantor

Hui Zheng.

## Provenance and peer review

Not commissioned, externally peer-reviewed.

## Data availability statement

Data used for analysis will be available upon reasonable request, which will be released in the Open Science Framework platform (https://osf.io/rq87j).

## Role of the funder/sponsor

The sponsors had no role in the design and conduct of the study, and they had no role in the decision process to submit the manuscript for publication.

## Supplementary Material
