# Supplementary figures and images for: Probiotics for the management of irritable bowel syndrome: a systematic review and three-level meta-analysis
Source: Int J Surg. 2023 Aug 10;109(11):3631–47. doi: 10.1097/JS9.0000000000000658 (PMC10651259; doi:10.1097/JS9.0000000000000658)

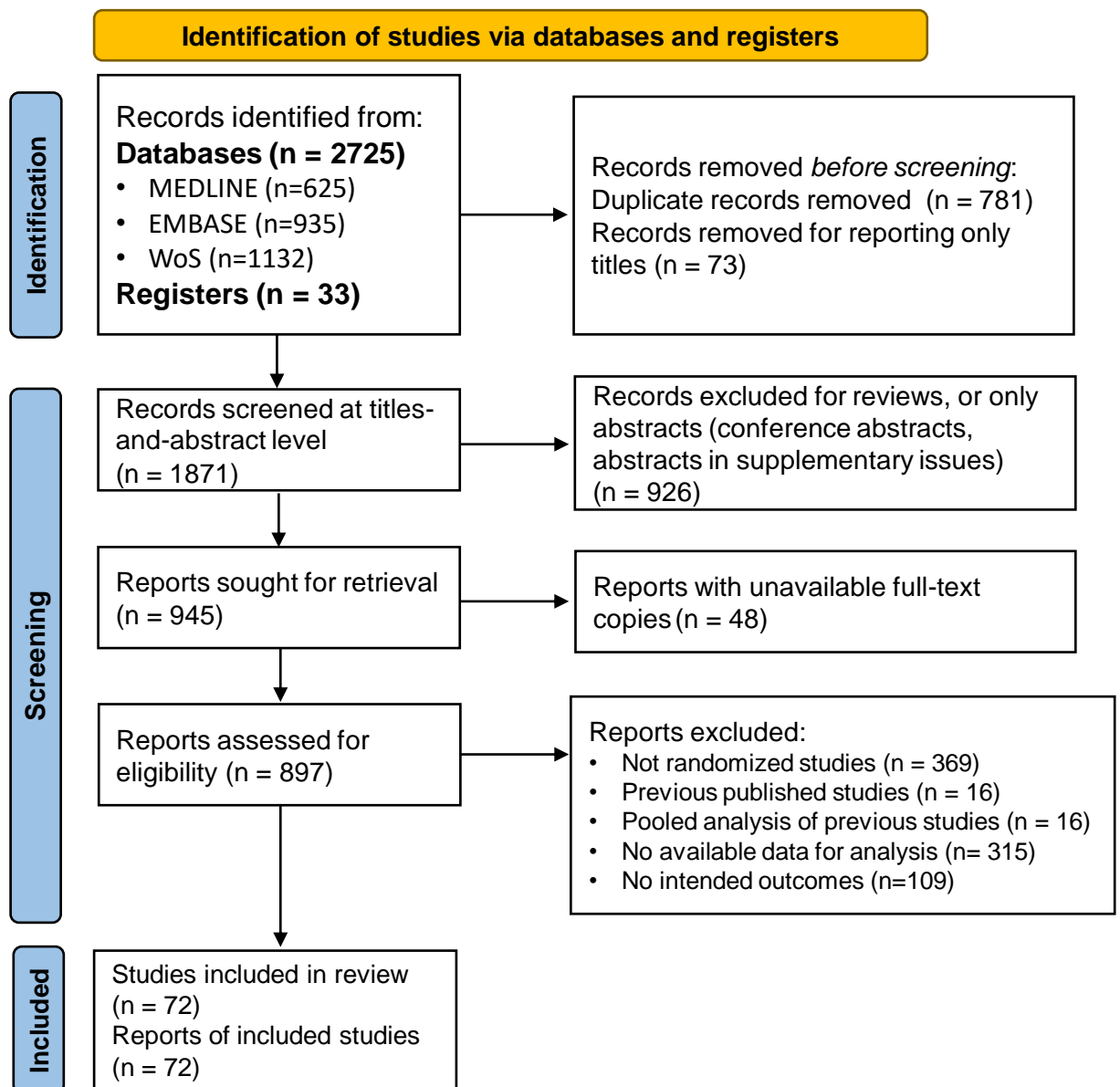

Supplement: Supplementary file 2 [file js9-109-3631-s002.pdf]
