# Supplementary material for: Probiotics for the management of irritable bowel syndrome: a systematic review and three-level meta-analysis
Source: Int J Surg. 2023 Aug 10;109(11):3631–47. doi: 10.1097/JS9.0000000000000658 (PMC10651259; doi:10.1097/JS9.0000000000000658)
Supplement: Supplementary file 4 [file js9-109-3631-s004.docx]

**Supplementary tables and figure**

[eTable 1. Search strategy for MEDLINE 2](#_Toc140223348)

[eTable 2. Search strategy for EMBASE 3](#_Toc140223349)

[eTable 3. Search strategy for Web of Science 5](#_Toc140223350)

[eTable 4. GRADE summary of findings 6](#_Toc140223351)

[eFigure 1. Risk of bias assessment 7](#_Toc140223352)

[eFigure 2. The funnel plot for global IBS symptoms 9](#_Toc140223353)

[eFigure 3. The funnel plot for abdominal pain 10](#_Toc140223354)

[eFigure 4. The funnel plot for quality of life 11](#_Toc140223355)

# eTable 1. Search strategy for MEDLINE

| **ID** | **Search strategy** |
| --- | --- |
| 1 | randomized controlled trial.pt. |
| 2 | controlled clinical trial.pt. |
| 3 | randomized.ab. |
| 4 | randomised.ab. |
| 5 | randomly.ab. |
| 6 | or/1-6 |
| 7 | limit 7 to humans |
| 8 | exp Irritable Bowel Syndrome/ |
| 9 | irritable bowel syndrome. ti,ab. |
| 10 | IBS. ti,ab. |
| 11 | IBS*. ti,ab. |
| 12 | or/9-11 |
| 13 | probiotic*.ti,ab. |
| 14 | Oligosaccharides.ti,ab. |
| 15 | Disaccharides.ti,ab. |
| 16 | Monosaccharides.ti,ab. |
| 17 | Polyols.ti,ab. |
| 18 | plantarum.ti,ab. |
| 19 | reuteri.ti,ab. |
| 20 | bifidobacterium.ti,ab. |
| 21 | saccharomyces.ti,ab. |
| 22 | infantis.ti,ab. |
| 23 | lactobacillus.ti,ab. |
| 24 | or/14-23 |
| 25 | 6 and 12 and 24 |

# eTable 2. Search strategy for EMBASE

| **ID** | **Search strategy** |
| --- | --- |
| 1 | ‘randomized Controlled Trial’/exp |
| 2 | ‘randomized Controlled Trials as Topic’/exp |
| 3 | ‘randomized controlled trial’:ab,ti |
| 4 | ‘controlled clinical trial’/exp |
| 5 | ‘controlled clinical trial’:ab,ti |
| 6 | (#1 OR #2 OR #3 OR #4 OR #5) AND [humans]/lim |
| 7 | ‘Irritable Bowel Syndrome’/exp |
| 8 | ‘irritable bowel syndrome’:ab,ti |
| 9 | ‘IBS’:ab,ti |
| 10 | ‘IBS*’:ab,ti |
| 11 | #7 OR #8 OR #9 OR #10 |
| 12 | ‘probiotic’:ab,ti |
| 13 | ‘Oligosaccharides’:ab,ti |
| 14 | ‘Monosaccharides’:ab,ti |
| 15 | ‘Polyols’:ab,ti |
| 16 | ‘plantarum’:ab,ti |
| 17 | ‘reuteri’:ab,ti |
| 18 | ‘bifidobacterium’:ab,ti |
| 19 | ‘saccharomyces’:ab,ti |
| 20 | ‘infantis’:ab,ti |
| 21 | ‘lactobacillus’:ab,ti |
| 22 | #7 OR #8 OR #9 OR #10 OR #11 |
| 23 | #6 AND #11 AND #22 |

# eTable 3. Search strategy for Web of Science

| **ID** | **Search strategy** |
| --- | --- |
| #1 | TS=(irritable bowel syndrome* or IBS) or TI=(irritable bowel syndrome* or IBS) or AB=(irritable bowel syndrome* or IBS) |
| #2 | TS=(randomized controlled trial or placebo or double blind) or TI=(randomized controlled trial or placebo or double blind) or AB=(randomized controlled trial or placebo or double blind) |
| #3 | TS=(probiotic or Oligosaccharides or Monosaccharides or Polyolsor or plantarum or reuteri or Bifidobacterium or saccharomyces or infantis or lactobacillus) or TI=(probiotic or Oligosaccharides or Monosaccharides or Polyolsor or plantarum or reuteri or Bifidobacterium or saccharomyces or infantis or lactobacillus) or AB=(probiotic or Oligosaccharides or Monosaccharides or Polyolsor or plantarum or reuteri or Bifidobacterium or saccharomyces or infantis or lactobacillus) |
| #4 | #3 AND #2 AND #1 |

# eTable 4. GRADE summary of findings

| **Probiotics compared with placebo for the treatment of irritable bowel syndrome (IBS)** | | | | |
| --- | --- | --- | --- | --- |
| **People:** Participants with irritable bowel syndrome  **Settings:** High- and middle-income countries  **Intervention:** Probiotics  **Comparison:** Placebo | | | | |
| **Outcomes** | **Relative effect (95%CI) ^1^** | **Number of studies** | **Certainty of the evidence (GRADE)*** | **Comments** |
| Global IBS symptoms | -0.55, 95%CI -0.76 to -0.34 | 63 | ⊕⊕⊖⊖  Low | A high risk of bias was noted in two of the included studies, so we downgraded the evidence from high to moderate. In addition, most of the included studies are with small sample sizes and under the risk of small-study effect, and we downregulated the evidence from moderate to low. |
| Abdominal pain | -0.89, 95%CI -1.29 to -0.5 | 48 | ⊕⊕⊖⊖  Low | A high risk of bias was noted in two of the included studies, so we downgraded the evidence from high to moderate. In addition, most of the included studies are with small sample sizes and under the risk of small-study effect, and we downregulated the evidence from moderate to low. |
| Quality of life assessment | 0.99, 95%CI 0.45 to 1.54 | 23 | ⊕⊕⊖⊖  Low | More than half of the included studies were classified with some concerns about the risk of bias assessment, so we downgraded the evidence from high to moderate. In addition, most of the included studies are with small sample sizes and under the risk of small-study effect, and we downregulated the evidence from moderate to low. |
| * GRADE Working Group grades of evidence  **High** = This research provides a very good indication of the likely effect. The likelihood that the effect will be substantially different^†^ is low.  **Moderate** = This research provides a good indication of the likely effect. The likelihood that the effect will be substantially different^†^ is moderate.  **Low** = This research provides some indication of the likely effect. However, the likelihood that it will be substantially different^†^ is high.  **Very low** = This research does not provide a reliable indication of the likely effect. The likelihood that the effect will be substantially different^†^ is very high.  ^†^ All three outcomes were measured and pooled by using the standardized mean difference (SMD), in which the cut-off points for small, medium, and large effects are 0.3, 0.5, and 0.8, respectively. | | | | |

# eFigure 1. Risk of bias assessment


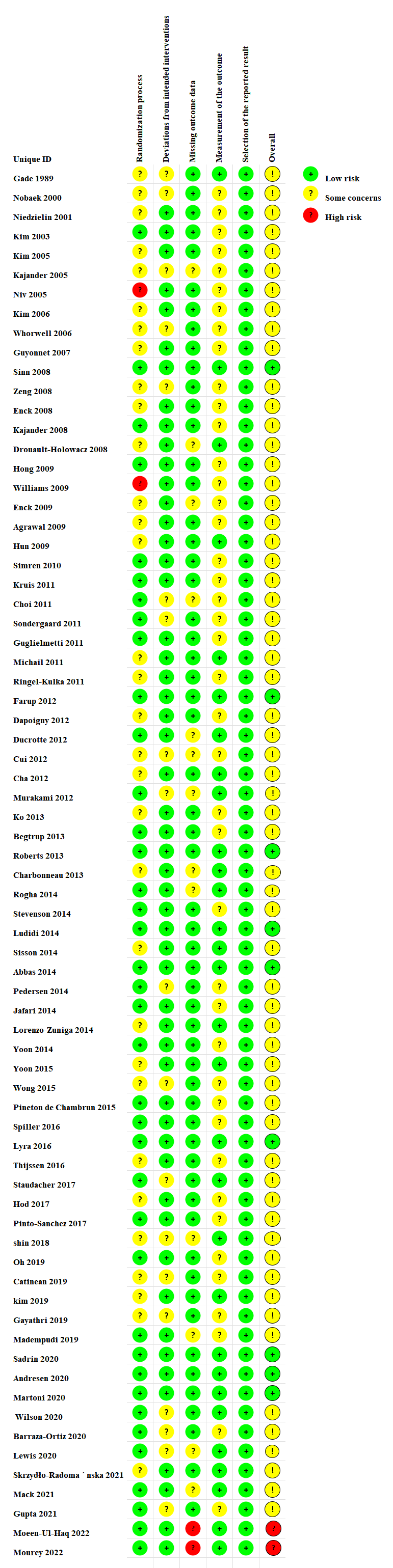


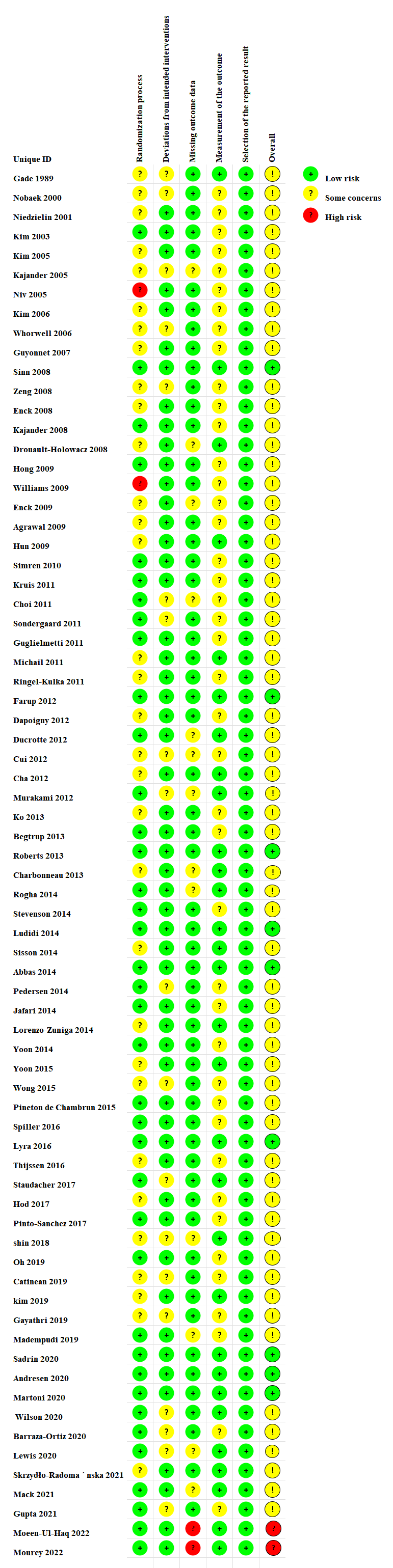


# eFigure 2. The funnel plot for global IBS symptoms


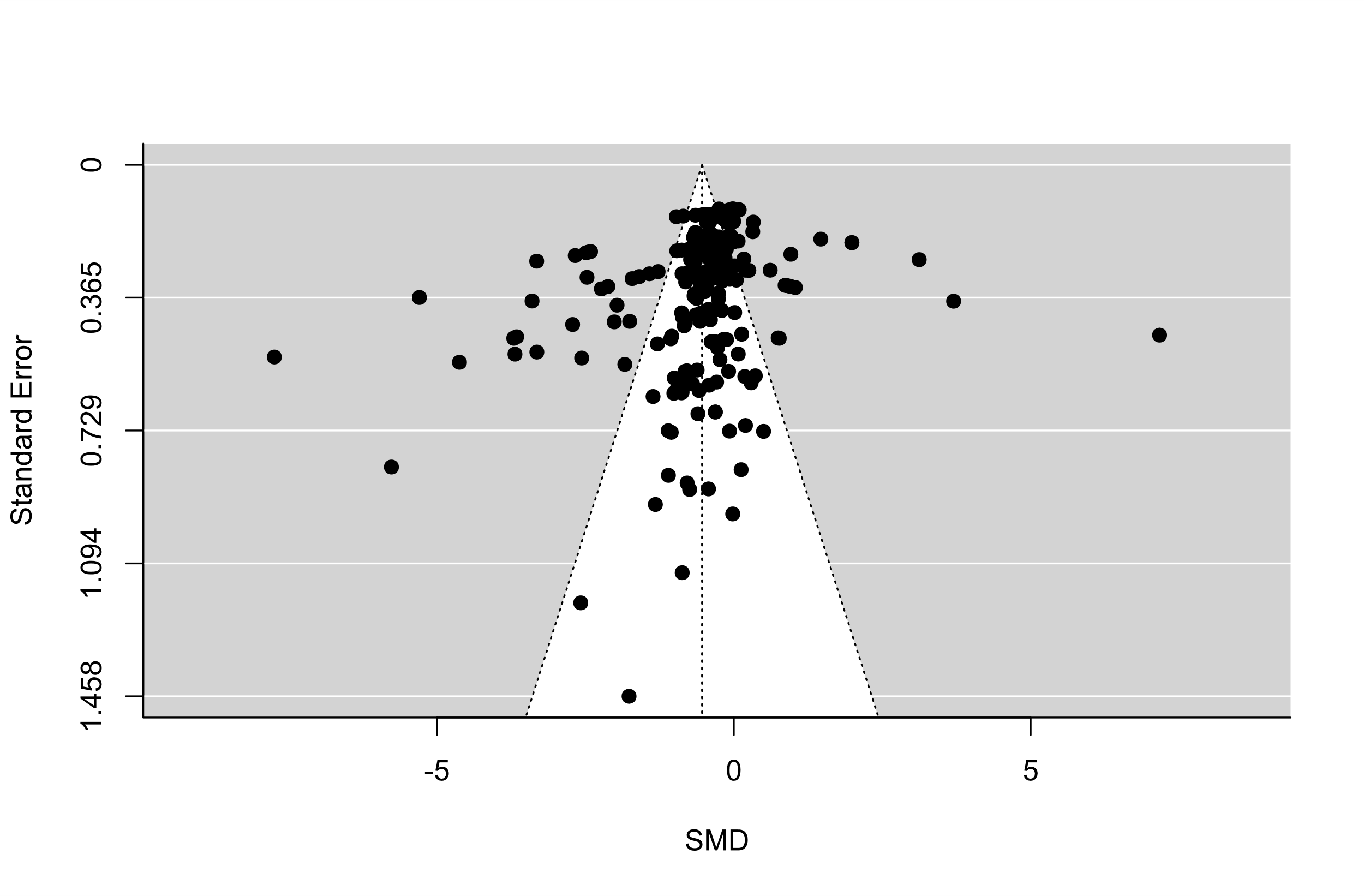


Abbreviations: SMD, standard mean difference.

Footnotes: Funnel plot was generated for visual assessment of asymmetric to find out any potential publication bias. This funnel plot shows symmetric signs and therefore excludes publication bias.

# eFigure 3. The funnel plot for abdominal pain


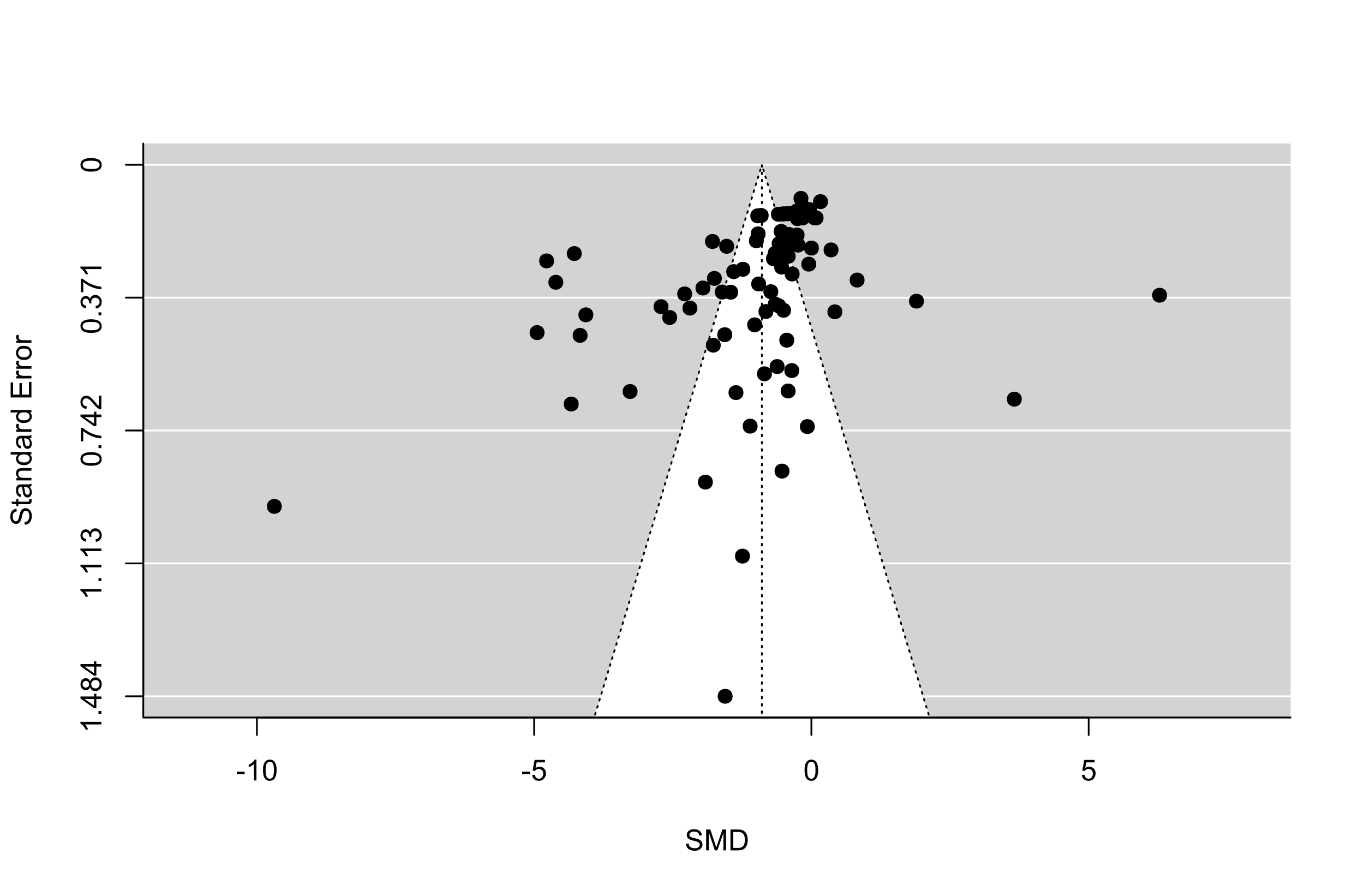


Abbreviations: SMD, standard mean difference.

Footnotes: Funnel plot was generated for visual assessment of asymmetric to find out any potential publication bias. This funnel plot shows symmetric signs and therefore excludes publication bias.

# eFigure 4. The funnel plot for quality of life


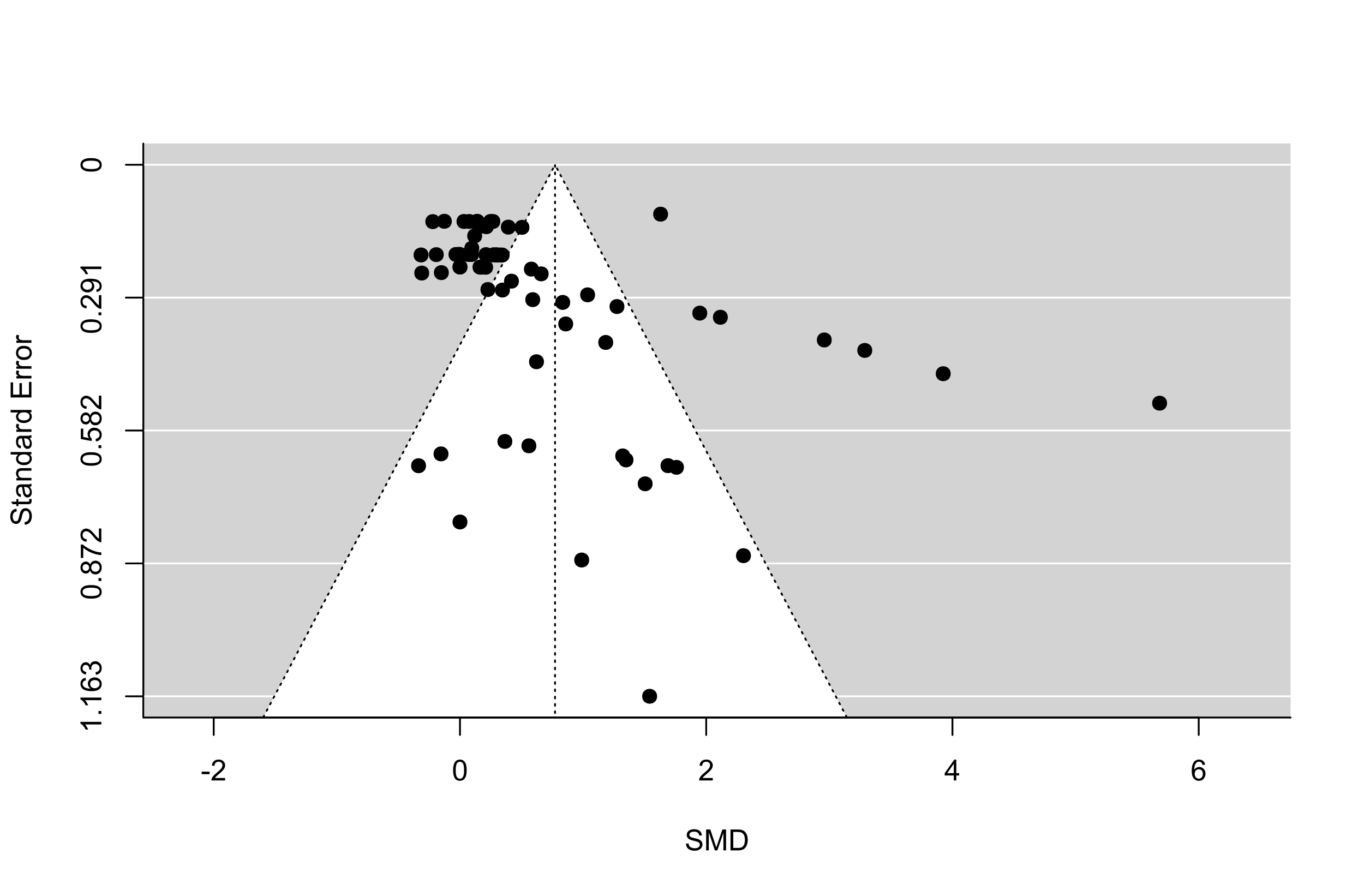


Abbreviations: SMD, standard mean difference.

Footnotes: Funnel plot was generated for visual assessment of asymmetric to find out any potential publication bias. This funnel plot shows asymmetric signs and therefore indicates potential publication bias.
